# Suppression of fat deposition in broiler chickens by (-)-hydroxycitric acid supplementation: A proteomics perspective

**DOI:** 10.1038/srep32580

**Published:** 2016-09-02

**Authors:** Mengling Peng, Jing Han, Longlong Li, Haitian Ma

**Affiliations:** 1 Key Laboratory of Animal Physiology and Biochemistry, College of Veterinary Medicine, Nanjing Agricultural University, Nanjing 210095, China; 2 School of Life Science and Technology, China Pharmaceutical University, Nanjing 210009, China

## Abstract

(-)-Hydroxycitric acid (HCA) suppresses fatty acid synthesis in animals, but its biochemical mechanism in poultry is unclear. This study identified the key proteins associated with fat metabolism and elucidated the biochemical mechanism of (-)-HCA in broiler chickens. Four groups (n = 30 each) received a diet supplemented with 0, 1000, 2000 or 3000 mg/kg (-)-HCA for 4 weeks. Of the differentially expressed liver proteins, 40 and 26 were identified in the mitochondrial and cytoplasm respectively. Pyruvate dehydrogenase E1 components (PDHA1 and PDHB), dihydrolipoyl dehydrogenase (DLD), aconitase (ACO2), a-ketoglutarate dehydrogenase complex (DLST), enoyl-CoA hydratase (ECHS1) and phosphoglycerate kinase (PGK) were upregulated, while NADP-dependent malic enzyme (ME1) was downregulated. Biological network analysis showed that the identified proteins were involved in glycometabolism and lipid metabolism, whereas PDHA1, PDHB, ECHS1, and ME1 were identified in the canonical pathway by Ingenuity Pathway Analysis. The data indicated that (-)-HCA inhibited fatty acid synthesis by reducing the acetyl-CoA supply, *via* promotion of the tricarboxylic acid cycle (upregulation of PDHA1, PDHB, ACO2, and DLST expression) and inhibition of ME1 expression. Moreover, (-)-HCA promoted fatty acid beta-oxidation by upregulating ECHS1 expression. These results reflect a biochemically relevant mechanism of fat reduction by (-)-HCA in broiler chickens.

Over the last few decades, the principle aim of poultry production in many countries has been to increase the growth rate of animals. However, modern broiler strains often tend to have excessive abdominal fat deposit^1^,^2^, which needs to be controlled, since it has a negative impact on poultry production, as evidenced by the increase in feed cost during rearing, decrease in the final meat quality, and the significant economic loss to poultry-processing plants^3^,^4^,^5^. Due to the economic concerns and consumer aversion to excess fat deposition, excess fat control and improvement of meat quality are important topics of research for poultry scientists.

(-)-Hydroxycitric acid [(-)-HCA], which is the major active ingredient present in the fruit rinds of *Garcinia cambogia*^6^,^7^, is known to promote weight loss^8^,^9^,^10^, increase the rate of glycogen synthesis^11^, suppress *de novo* fatty acid synthesis^12^,^13^ and increase lipid oxidation^14^,^15^,^16^. Recently, our laboratory also found that *Garcinia cambogia* extracts could attenuate fat accumulation through regulation of lipolysis gene expression via the adiponectin-AMPK signaling pathway in a rat obesity model induced by a high-fat diet^17^. Further, previous studies have shown that in animals and humans, (-)-HCA is a potent inhibitor of ATP-citrate lyase^18^,^19^, which catalyzes the cleavage of citrate to oxaloacetate and acetyl-CoA and eventual limits the availability of the acetyl-CoA units required for fatty acid synthesis and lipogenesis^10^,^20^. However, the underlying biochemical mechanism is not well understood, especially the effects of (-)-HCA in broiler chickens.

Lipid metabolism in poultry differs from that in mammals, with the liver being the main organ involved in metabolic activity in poultry^21^,^22^. In poultry, the catabolism of fatty acids (β-oxidation) primarily occurs in the mitochondria, whereas fatty acids are synthesized in the cytoplasm of hepatocytes^22^. The proteomics approach is a powerful tool for studying biological mechanisms^23^,^24^. Further, a global protein expression analysis of the liver would aid in the identification of differentially expressed proteins involved in lipid metabolism and provide new insight into the mechanism of fat deposition in broiler chickens.

Dietary supplements of *Garcinia cambogia* extracts, a potential therapy for decreasing fat deposition, may be a practical way of reducing excessive carcass fat in poultry. The current study was designed to explore the effect of (-)-HCA supplementation on the hepatic expression (mitochondrial and cytoplasmic) of lipid metabolism-related proteins/enzymes in broiler chickens. The aim was to identify the different proteins that are involved in lipid metabolism and to gain a better understanding of the biochemical mechanism of (-)-HCA regulation of fat deposition in poultry.

## Material and Methods

### Materials and reagents

Isoelectric pH gradient (IPG) strips (pH 3.0–10.0; NL, 17 cm), urea, pharmalyte (pH 3–10), glycerol (87% w/w), Tris (electrophoresis grade), 1,2-di(dimethylamino)ethane (TEMED; electrophoresis purity reagent), acrylamide (40% solution; acrylamide-to-bisacrylamide ratio, 37.5:1), 3-[(3-cholamidoprpyl) dimethylammonio]-1-propanesulfonate (CHAPS; electrophoresis grade), thiourea (ACS grade), dithiothreitol (DTT, electrophoresis grade), iodoacetamide (electrophoresis grade), mineral oil, Coomassie G-250 stain and low-melting-point agarose were obtained from Bio-Rad. Animal cell/tissue quality purified mitochondria isolation kits were purchased from Genmed Scientifics, and the enzyme activity assay kits were purchased from Nanjing Jiancheng Biotechnology Institution. High-purity water prepared from the Milli-Q gradient water purification system (Millipore) was used for all the experiments in this study.

### Garcinia cambogia extracts

*Garcinia cambogia* extracts were obtained from An Yun Co. Ltd. (Zhengzhou, China). The *G.arcinia cambogia* extracts contained 56–58% (-)-HCA, as well as 12–14% cellulose, 5.5–6% α-d-melibiose, 2.5–3% β-d-lactin, 1.5–2% d-mannopyranose, 11–12% oxophenic acid, 2–3% octadecyl alcohol, 3.5–4% coenzyme A and 1.5–2% inorganic elements.

### Animals and treatment

A total of 120 one-day-old broiler chickens (Ross 308) were obtained from Jiangsu Wuxi chicken breeding company (Wuxi, China). The birds were weighed and allocated to four treatment groups, each of which included three replicates of 10 birds. The broiler chickens were fed the same basal diets from 1 to 49 d (including the starter phase [days 1–21] and finisher phase [days 22–49]). The dietary nutrient levels were in compliance with the nutrient requirements for broiler chickens recommended by the National Research Council^25^. All animal handling procedures were performed in strict accordance with guide for the Care and Use of Laboratory Animals central of the Nanjing Agricultural University (Nanjing, China), and the protocol was approved by the Institutional Animal Care and Use Committee of the Nanjing Agricultural University (Nanjing, China).

During the finisher phase, the four groups of chickens were supplemented with *Garcinia Cambogia* extracts at concentrations of 0, 25, 50 and 75 g/kg diet, which were equivalent to 0 mg/kg, 1000 mg/kg, 2000 mg/kg and 3000 mg/kg (-)-HCA respectively. In the starter phase, the broiler chickens were housed in lighted coops with constant lighting and water was provided continuously. The temperature was set at 32 °C for the initial 5 d and then gradually reduced according to normal management practices, until a temperature of 22 °C was reached. The broilers were floor-reared under natural lighting during the finisher phase. At the end of the experiment, birds were randomly selected, deprived of feed for 12 h, weighed and sacrificed. The liver was collected and snap-frozen in liquid nitrogen. Frozen tissues were stored at −80 °C until analysis.

### Sample preparation

The cytoplasmic and mitochondrial fractions were separated by differential centrifugation and purified using a two-step gradient according to the instructions provided in the kit. In this extraction process, the post-nuclear supernatant and mitochondrial fractions were assayed for mitochondrial marker enzymes according to previously reported methods^26^.

### Two-dimensional electrophoresis

The protein concentration of each of the final supernatants was measured using the Bradford assay^27^ with bovine serum albumin (BSA) as the standard. Protein extracts (850 μg) were separated by isoelectrophoresis (IEF) using IPG strips (pH 3.0–10.0; NL, 17 cm) in the protean system (Bio-Rad) at 20 °C. Focusing was performed for 1 h at 250 V, 1 h at 500 V, 1 h at 2000 V, and 2.5 h at 8000 V; it was then held at 8000 V until the total potential was at least 60,000 V. After the IEF run was complete, the strips were removed and equilibrated with gentle shaking in two subsequent steps for 15 min each in 5 mL equilibration buffer (0.05 M Tris-HCl [pH 8.8], 6 M urea, 30% glycerol, and 2% SDS) containing an additional 0.1 mg DTT in the first step and 0.1 mg iodoacetamide in the second step. The second dimension was run on a 12.5% polyacrylamide SDS gel using the Multiphor system (Amersham Biosciences). Two-dimensional electrophoresis (2-DE) for each sample was repeated three times.

Neuhoff’s colloidal Coomassie Blue G-250 staining was performed according to the method described by Giovanni *et al*.^28^. Stained gels were scanned and analyzed using the PDQuest 2-D analysis software version 8.0 (Bio-Rad). After alignment, spots between gels were first automatically matched, and the matched spots were then re-examined manually to ensure accuracy. Only spots with quality >50 and expression difference >2 were chosen for further analysis. Spot quantity normalization was conducted in the ‘total quantity of valid spots’ mode.

### MALDI-TOF MS analysis

In-gel trypsin digestion of protein spots and matrix-assisted laser desorption/ionization-time of flight mass spectrometry (MALDI-TOFMS; Reflex III, Bruker-Daltonics) were performed using procedures described by Chen *et al*.^26^. MS fingerprinting data searches were performed by using MS-fit search engines (http://prospector.ucsf.edu) against the NCBInr database for *Gallus gallus*, with the parameters set to trypsin digestion, two missed cleavages, complete modification of iodoacetamide (Cys), partial modification of methionine oxidation, protein mass (±20% of the observed protein mass, pI), and mass tolerance for mono-isotopic data of 100 ppm. The protein was considered to have been identified when there were at least four matching peptides and >20% sequence coverage. The functions of the proteins identified through MS fingerprinting data were annotated by querying against the protein knowledge database UniprotKB (http://www.uniprot.org/uniprot/).

### Functional annotation of identified proteins

Gene ontology (GO) is widely used to describe protein function in a standardized format^29^. GO analysis of the identified proteins was performed using the Database for Annotation^30^. The GoMiner tool was used to group proteins according to their biological process and molecular function, as this tool provides an overview of the main biological processes in which these proteins participate. We also performed pathway enrichment analysis using the Kyoto Encyclopedia of Genes and Genomes (KEGG) pathway maps^31^. In addition, Ingenuity Pathway Analysis (IPA) was also used to build the interaction network and canonical pathways.

## Results

### Purity analysis of isolate mitochondrial fraction

**Figure 1:**
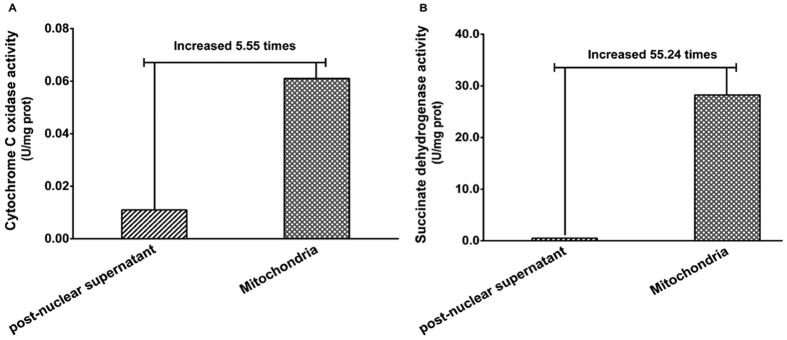
The activities of mitochondria-specific marker enzymes. (**A**) Cytochrome C oxidase activity; (**B**) Succinate dehydrogenase activity. Values represent the activity of the marker enzymes in the post-nuclear supernatant and mitochondrial fraction, expressed as the change in absorbance per hour per milligram of protein.

The purity of mitochondrial proteins was evaluated by assessing the activity of mitochondrial -specific marker enzymes. As shown in Fig. 1, the activity of cytochrome C oxidase (a mitochondrial marker enzyme) in the mitochondrial fraction was 5.55 times higher than that in the post-nuclear supernatant (Fig. 1A). Succinate dehydrogenase is another marker enzyme that is also the only enzyme that binds to the inner mitochondrial membrane in cells. Our results showed that the succinate dehydrogenase activity in the mitochondrial fraction was 55.24 times higher than that in the post-nuclear supernatant (Fig. 1B). These results indicate that the mitochondrial fraction was effectively enriched, and therefore, the mitochondrial samples could be used for protein analysis by 2-DE gel electrophoresis.

### Proteome profile of the liver in broiler chickens fed (-)-HCA supplements

**Figure 2:**
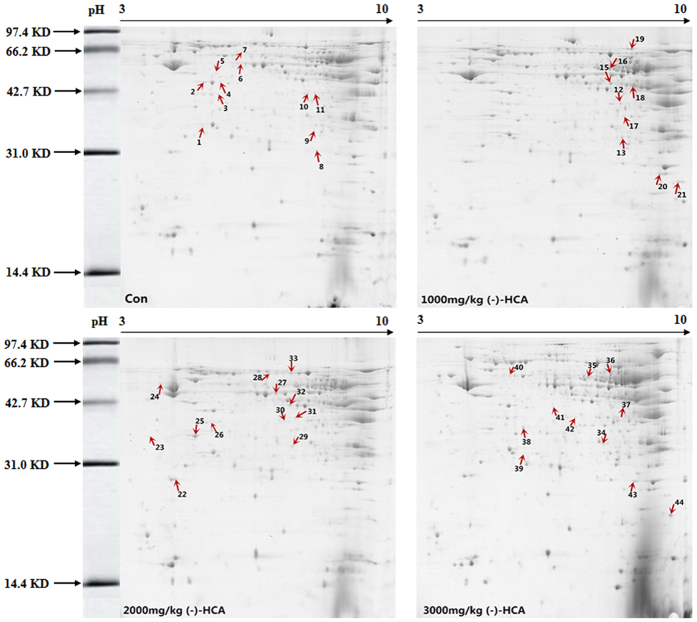
Impact of (-)-HCA on the protein/enzyme profile of the liver mitochondrial fraction in broiler chickens. The arrows in every gel indicate differentially expressed proteins/enzymes that exhibit at least a 2.0-fold difference between the control group and (-)-HCA-treated samples. The significance of the altered spots was assessed by matrix-assisted laser desorption/ionization time of flight (MALDI-TOF) mass spectrometry, and the identified proteins/enzymes are listed in Table 1.

**Figure 3:**
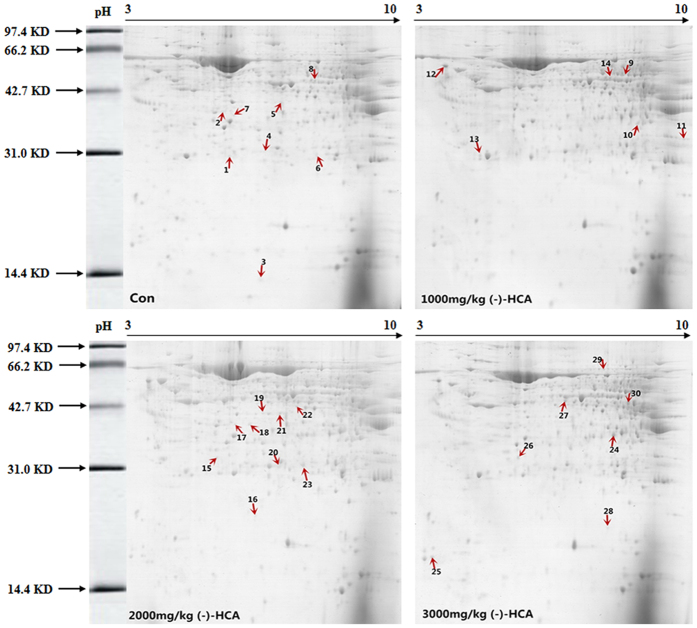
Impact of (-)-HCA on the protein/enzyme profile of the liver cytoplasmic fraction in broiler chickens. The arrows in every gel indicate differentially expressed proteins/enzymes that exhibit at least a 2.0-fold difference between the control group and (-)-HCA-treated samples. The significance of the altered spots was assessed using matrix-assisted laser desorption/ionization time of flight (MALDI-TOF) mass spectrometry, and the identified proteins/enzymes are listed in Table 2.

Proteomics analysis was performed to investigate differentially expressed proteins in broiler chickens after (-)-HCA supplementation, and images of the representative 2D gels are shown in Figs 2 and 3. In the 1000 mg/kg, 2000 mg/kg, and 3000 mg/kg (-)-HCA treatment groups, 21, 37, and 17 mitochondrial protein spots, respectively, and 17, 17 and 12 cytoplasmic protein spots, respectively, showed significantly different expression. The distribution of the molecular weight of the proteins was found to be approximately between 14.4 and 97.4 kDa. After auto-matching and a manual quality check of the detected spots, it was found that over 75% of the spots matched. Generally, the overall distribution of proteins in the 2-DE gels was similar in the four groups. Protein spots that showed >2-fold difference in expression between the (-)-HCA and control groups were selected for further analysis. In all, 40 protein spots in the mitochondrial samples and 26 protein spots in the cytoplasmic samples were cut out from the Coomassie-stained 2D gels for in-gel digestion and analyzed by MALDI-TOF-MS. Protein identification using the MS fingerprint data was conducted by querying the NCBInr protein databases. Among the 40 identified mitochondrial proteins, 31 were upregulated and 9 were downregulated in the (-)-HCA treatment group (the marked spots in Fig. 2). Moreover, among the 26 identified cytoplasmic proteins, 21 were upregulated and 5 were downregulated (marked spots in Fig. 3). The protein spots were labeled numerically, and correspond to the proteins listed in Tables 1 and 2. A relatively high proportion of proteins were upregulated or downregulated, which indicates that (-)-HCA supplementation may lead to rather significant changes in the metabolism of broiler chickens.

### Functional annotation of the identified proteins

**Figure 4:**
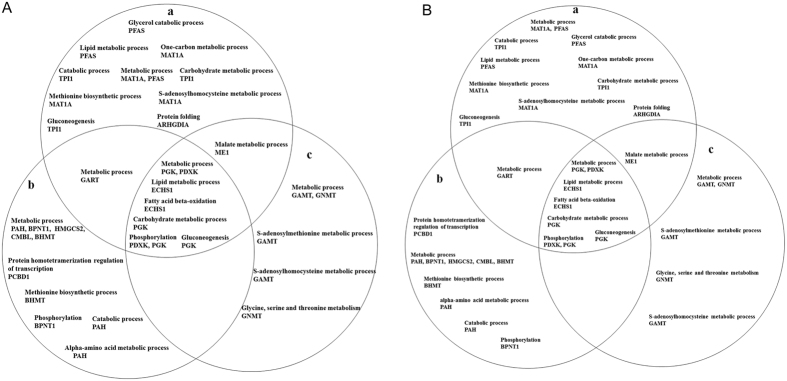
Schematic Venn diagram of the protein spots identified. Panels (**A,B**) depict differentially expressed proteins in the mitochondrial and cytoplasmic, respectively. (a) 1000 mg/kg (-)-HCA group, (b) 2000 mg/kg (-)-HCA group, (c) 3000 mg/kg (-)-HCA group. The diagram was drawn after analysis of the identified proteins using the GoMiner tool. Based on their categorization, the differentially expressed proteins were selected for the functional analysis.

To explore the biological functions of the identified proteins, we performed a computational study using GoMiner. With the GoMiner tool, the identified proteins were clustered into hierarchical categories based on their biological processes and molecular functions. The results showed that the identified mitochondrial proteins were involved in the metabolic process (PDHB, SUOX, NDUFA10, NDUFS3, HGD, ALDH2 and ECH1), the tricarboxylic acid cycle (ACO2, DLST, SDHA and PDHB), the catabolic process (ALDH6A1, PRDX3, CAT and DYPS), fat cell differentiation (ALDH6A1), gluconeogenesis (PC, PDHA1, ALDH2, DLD, PCK2 and PDHB), acetyl-CoA biosynthesis (PDHA1 and PDHB), mitochondrial electron transport (UQCRC2 and DLD), the carbohydrate metabolic process (PC and PCK2), the malate-aspartate shuttle (SLC25A13), the response to lipopolysaccharide (PRDX3) and the metabolism of some amino acids (Fig. 4A). Similarly, the identified cytoplasmic proteins were involved in the metabolic process (PGK, GART, MAT1A, ME1, PFAS, PAH, BPNT1, GAMT, PDXK, HMGCS2, CMBL, GNMT and BHMT), the catabolic process (TPI1 and PAH), gluconeogenesis (PGK and TPI1), the malate metabolic process (ME1), fatty acid beta-oxidation (ECHS1), the lipid metabolic process (PFAS) and phosphorylation (PDXK, PGK and BPNT1) (Fig. 4B). A number of upregulated or downregulated proteins were found to be associated with lipid metabolism and glycometabolism, which indicates the possible involvement of these proteins in the regulation of fat deposition by (-)-HCA in broiler chickens.

### Biological network analysis of the identified proteins

To elucidate the metabolic pathways that the identified proteins are involved in, KEGG pathway enrichment analysis was used to analyze the differentially expressed proteins. The results showed that the identified proteins were mainly involved in the citrate cycle, glycolysis/gluconeogenesis, pyruvate metabolism, fatty acid metabolism, beta-alanine metabolism, oxidative phosphorylation and the PPAR signaling pathway. Importantly, most of differentially expressed proteins were involved in metabolic pathways (ACO2, ALDH2, ALDH6A1, ATP5H, CPOX, DLD, DLST, DYPS, GLUL, HGD, NDUFA10, NDUFS3, NDUFS8, PC, PCK2, ADSL, BHMT, BPNT1, CMBL, ECHS1, GAMT, GART, HMGCS2, MAT1A, ME1, PAH, PGK, PDXK, PFAS, TPI1, PDHA1, PDHB, RGN, SDHA, SUCLG2, TST and UQCRC2).

**Figure 5:**
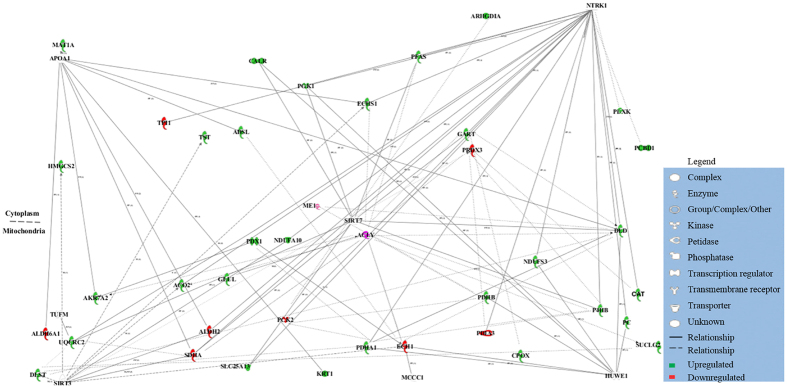
Canonical pathway built using 66 differentially expressed proteins. The network was created using Ingenuity Pathway Analysis. Red indicates that the expression of the corresponding protein was downregulated, and green indicates that the expression of the corresponding protein was upregulated in the liver of broiler chickens that were given (-)-HCA. The full and dashed lines represent direct and indirect interactions between the proteins, respectively. Network shapes are explained in the legend.

Interaction networks of the identified proteins were generated using IPA, which revealed the potential networks and connections between the identified proteins. Both mitochondrial and cytoplasmic proteins were found to participate in the metabolic pathways via a common interaction network. Thus, we drew the canonical networks together with the main canonical pathway and identified the proteins involved in metabolism (Fig. 5). This network comprised 53 proteins, 35 of which were differentially expressed between the control group and (-)-HCA groups. Of the differentially expressed proteins, 27 were upregulated (MAT1A, HMGCS2, DLST, UQCRC2, AKRTA2, ADSL, TST, ACO2, SLC25A13, GLUL, PDX1, NDUFA10, KRT1, PDHA1, CPOX, SUCLG2, PC, CAT, PDHB, NDUFS3, DLD, PCBD1, GART, ARHGDIA, ECHS1, PFAS and CALR, shown in green), and 8 were downregulated (ALDH6A1, TPI1, PRDX3, ALDH2, SDHA, PCK2, ECH1 and PDIA3, shown in red). These proteins were mainly associated with the metabolic process, the tricarboxylic acid cycle, the catabolic process, brown fat cell differentiation, gluconeogenesis, the acetyl-CoA biosynthetic process, the malate-aspartate shuttle, response to lipopolysaccharide, fatty acid beta-oxidation, the lipid metabolic process and phosphorylation. However, it should be noted that not all the differentially expressed proteins involved in the biological network were identified.

## Discussion

Although many studies had reported that (-)-HCA promote weight loss^9^,^10^, suppresses *de novo* fatty acid synthesis^13^,^32^, increases lipid oxidation^14^,^16^ and enhances energy expenditure^33^,^34^, while the precise biochemical mechanism is not full clear. Unlike mammalian, liver is the main organ of lipid metabolism in poultry^21^,^22^. The present study presents the global liver protein profile of broiler chickens after dietary (-)-HCA supplementation: 40 and 26 differentially expressed proteins were identified in the mitochondrial and cytoplasmic respectively. These differentially expressed proteins can provide detailed biological information on the action of (-)-HCA in broiler chickens.

Our results showed that the NDUFS3, NDUFS8 and NDUFA10 protein expression levels were increased in the liver of broiler chickens after (-)-HCA supplementation. NDUFS3 and NDUFA10 are important subunits of NADH dehydrogenase: NDUFS3 plays a vital role in the proper assembly of complex I^35^,^36^ and NDUFA10 transfers electrons from NADH to ubiquinone in the respiratory chain^37^. Our results also showed that the UQCRC2 protein expression level was enhanced in all the (-)-HCA groups. UQCRC2 is a core protein that is required for the assembly of the ubiquinol-cytochrome c reductase complex (complex III), which is part of the mitochondrial respiratory chain^38^. Further, our results showed that (-)-HCA increased the ATP5H protein expression level in the liver of broiler chickens. As a subunit of ATP synthase, ATP5H plays an important role in the transfer of hydrogen ions from one side of a membrane to the other^39^. Our results also showed that the succinate dehydrogenase flavoprotein subunit (SDHA) protein expression level was downregulated while the succinyl-CoA ligase subunit beta (SUCLG2) protein expression level was upregulated in broiler chickens that were given (-)-HCA. Succinate dehydrogenase is the only enzyme that participates in both the citric acid cycle and the electron transport chain^40^. Based on the upregulated expression of NDUFS3, NDUFA10, UQCRC2, ATP5H and SUCLG2, we think that (-)-HCA treatment may accelerate energy metabolism in broiler chickens, and that this may be achieved mainly through the NADH respiratory chain. This seems to be in agreement with previous studies which reported that the suppressive effect of *G. cambogia* extracts on body weight gain depends on the improvement of energy expenditure^33^,^34^.

Our results showed that pyruvate dehydrogenase E1 component subunit alpha (PDHA1) and beta (PDHB) protein expression levels were increased in the liver of broiler chickens. The pyruvate dehydrogenase complex is a nuclear-encoded mitochondrial matrix multi-enzyme complex that acts as the primary link between the tricarboxylic acid cycle and fatty acid synthesis^41^. In addition, the expression of dihydrolipoyl dehydrogenase (DLD), which is another important enzyme of the pyruvate dehydrogenase complex, was enhanced in the liver of broiler chickens after (-)-HCA supplementation. Aconitase (ACO2,) which is known to be involved in the citrate cycle^42^, was also upregulated in the liver of broiler chickens given (-)-HCA. Previous studies have shown that the ACO2 expression level is upregulated to improve the efficiency of the tricarboxylic acid cycle when energy is required in the body^42^. In addition, the protein expression of the α-ketoglutarate dehydrogenase complex (DLST), which is a mitochondrial enzyme that plays a key role in the citric acid cycle, was also increased in the liver of broiler chickens after (-)-HCA treatment. These results indicate that (-)-HCA decreases fatty acid synthesis through promotion of the tricarboxylic acid cycle by upregulation of PDHA1, PDHB, DLD, ACO2 and DLST protein expression. In agreement with these findings, other studies have also reported that (-)-HCA can inhibit de novo fatty acid synthesis in animals and humans^12^,^13^. Pyruvate carboxylase (PC) is a mitochondrial protein that acts as a cross-link between carbohydrate and lipid metabolism^43^. PC can catalyze the reversible carboxylation of pyruvate to form oxaloacetate, while citrate synthase catalyzes the condensation reaction between acetyl-CoA and oxaloacetate to form citrate^44^. The oxaloacetate formed can be used for gluconeogenesis by phosphoenolpyruvate carboxykinase (PEPCK), which is catalyzed in the liver or returned to the mitochondria as malate^45^. Our results showed that the PC protein expression level was increased, while the PEPCK protein expression level was decreased in broiler chickens after (-)-HCA treatment. This result implied that in broiler chickens fed (-)-HCA, oxaloacetate might get converted into citrate rather than glucose via gluconeogenesis.

Previous studies have shown that (-)-HCA is a potent inhibitor of ATP-citrate lyase^19^ in animals and humans. This enzyme catalyzes the cleavage of citrate to oxaloacetate and acetyl-CoA and eventually limits the availability of acetyl-CoA units required for fatty acid synthesis and lipogenesis^10^,^20^. However, the underlying biochemical mechanisms are not well understood; in particular, there is no detailed information available on the effect of (-)-HCA in broiler chickens. In this study, no significant changes were observed in the ATP-citrate lyase expression levels in the broiler chickens given (-)-HCA supplements, which contradicts previous reports that (-)-HCA is an inhibitor of this enzyme. This discrepancy may have occurred because the ATP-citrate lyase protein expression level rather than its activity was detected in the present study.

In the avian liver, most of the NADPH used by fatty acid synthase to catalyze the synthesis of palmitate is generated by malic enzyme (ME1)^46^. It has been reported that level of hepatic malic enzyme is positively correlated with the rate of fatty acid synthesis, the percentage of body fat, and the percentage of abdominal fat in chicks^47^. In the present study, (-)-HCA treatment increased the NADP-dependent ME1 protein expression level in broiler chickens. Thus, we think that the suppressive effect of (-)-HCA on fat deposition was brought about via a decrease in the level of cytosolic acetyl-CoA and NADPH, which are required for fatty acid synthesis, *via* inhibition of ME1 protein expression.

Our present results showed that expression of the enoyl-CoA hydratase short chain 1 (ECHS1) protein, which is active in the beta-oxidation pathway of fatty acid metabolism^48^, was upregulated in the liver of broiler chickens after (-)-HCA treatment. The downregulation of ECHS1 has been reported to contribute to lipid accumulation in the liver^49^,^50^. Further, our results showed that the phosphoglycerate kinase 2 (PGK2) protein expression level was enhanced in broiler chickens after (-)-HCA treatment. Glycerol can be converted into α-phosphoglycerate via the action of PGK, after which it enters the gluconeogenic pathway in the liver^51^. Thus, the above data indicate that another important effect of (-)-HCA regulation on abdominal fat deposition was the promotion of fatty acid beta-oxidation *via* upregulation of ECHS1 protein expression in broiler chickens.

KEGG pathway and canonical pathway analysis showed that the differentially expressed proteins were mainly associated with metabolism, both lipid metabolism and glycometabolism, such as PCK2, PGK1, PRDX3, GART, PDX1, NDUFS3, PDHB, ECH1, ALDH2, PDHA1, UQCRC2, CALR and PFAS. These proteins have been previously reported to be involved in lipid metabolism and glycometabolism^52^,^53^,^54^, and activation of these proteins results in changes in lipid metabolism and glycometabolism in the tricarboxylic acid cycle, gluconeogenesis, fatty acid beta-oxidation and fat cell differentiation. Indeed, two of the proteins upregulated in the (-)-HCA-treated groups were PDHA1 and PDHB, which are the two central proteins identified in the canonical pathway by IPA. Both of these proteins participate in the citrate cycle, pyruvate metabolism, oxidative phosphorylation and glycolysis/gluconeogenesis. These results also indicate that (-)-HCA promotes the tricarboxylic acid cycle by up regulating PDHA1, PDHB, ACO2 and DLST protein expression. ECHS1 and ME1 expression was found to be upregulated and downregulated, respectively, in the network. They are involved in fatty acid synthesis and the beta-oxidation pathway. These findings indicate that (-)-HCA could affect lipid metabolism and glycometabolism via its effects on the expression of the key proteins in the network, such as some of the central proteins in the canonical pathway (PDHA1, PDHB, ECHS1 and ME1). Taken together, our findings show that 53 of the identified proteins were associated with the progression of metabolism observed with (-)-HCA treatment.

In conclusion, the present data indicate that (-)-HCA inhibited fat deposition in broiler chickens mainly via these two mechanisms: (1) inhibition of fatty acid synthesis *via* decrease in the supply of acetyl-CoA, which was mainly achieved by promotion of the tricarboxylic acid cycle (upregulation of PDHA1, PDHB, ACO2, DLST protein expression) and inhibition of ME1 protein expression; (2) promotion of fatty acid beta-oxidation by upregulation of ECHS1 protein expression. Further, IPA showed that these differentially expressed proteins were involved in glycometabolism and lipid metabolism, including PDHA1, PDHB, ECHS1, and ME1 in the canonical pathway, which reflected a biochemically relevant (-)-HCA-induced fat reduction mechanism in broiler chickens. In addition, global protein expression analysis could provide a basis for further studying the mechanism of (-)-HCA in the inhibition of body weight gain and fat deposition in animals.

## Additional Information

**How to cite this article**: Peng, M. *et al*. Suppression of fat deposition in broiler chickens by (-)-hydroxycitric acid supplementation: A proteomics perspective. *Sci. Rep.* **6**, 32580; doi: 10.1038/srep32580 (2016).

## Figures and Tables

**Table 1:** Differentially-expressed proteins in mitochondrial of liver in broiler chickens with (-)-HCA treatment identified by PMF query$.

Spot No.	Gene Ontology	Protein name	Accession No.	Exp. MW(KDa)/pI	Matched peptides	Score	Expression change treated with (-)-HCA#		
							1000 mg/Kg	2000 mg/Kg	3000 mg/Kg
1	NDUFA10	NADH dehydrogenase [ubiquinone] 1 alpha subcomplex subunit 10	gi|71895153	41.43/6.15	2	189	↑	↑	/
2	DYPS	dihydropyrimidinase	gi|363731013	58.03/5.73	2	147	↑	↑	/
3	NDUFS3	NADH dehydrogenase [ubiquinone] iron-sulfur protein 3	gi|226437575	29.23/6.55	7	449	↑	↑	↑
4	ACO2	aconitate hydratase	gi|45383738	85.79/8.05	4	260	↑	↑	
5	Prss1	protease, serine, 1 precursor	gi|16716569	26.13/4.75	1	70	↑	/	↑
6	N/A	Chain A, Crystal Structure Of The First Active Autolysate Form Of The Porcine Alpha Trypsin	gi|1942351	13.297.83	1	126	↓	/	↓
7	HGD	homogentisate 1, 2-dioxygenase	gi|50729534	49.476.35	1	57	↓	/	/
8	PC	pyruvate carboxylase	gi|45383466	127.25/6.26	7	479	↑	↑	/
9	EETI-II	Chain E, Complex Of Eeti-II With Porcine Trypsin	gi|157878102	24.17/8.26	1	78	↑	/	/
10	UQCRC2	cytochrome b-c1 complex subunit 2, mitochondrial-like	gi|118098350	48.58/9.04	4	331	↑	↑	↑
11	N/A	Chain B, Refined 1.8 Angstroms Resolution Crystal Structure Of Porcine Epsilon-Trypsin	gi|999627	8.82/6.67	1	137	↑	↑	↑
12	AKR7A2	aflatoxin B1 aldehyde reductase member	gi|118101125	36.62/6.76	2	110	↑	/	/
13	PDHA1	pyruvate dehydrogenase E1 component subunit alpha, somatic form, mitochondrial precursor	gi|60302740	44.44/8.19	3	129	↑	↑	/
14	ATP5H	ATP synthase subunit d, mitochondrial isoform 1	gi|118099965	18.34/8.73	2	85	↑	↑	/
15	MUC5AC	mucin-5AC, partial	gi|332267570	92.18/6.33	1	133	↑	↑	↑
16	N/A	Chain A, Chicken Cytochrome Bc1 Complex Inhibited By An Iodinated Analogue Of The Polyketide Crocacin-D	gi|196049775	49.44/5.95	2	117	↑	↑	↑
17	ALDH2	aldehyde dehydrogenase, mitochondrial isoform 2	gi|363739855	56.79/7.92	3	217	↓	↑	/
18	PDIA3	protein disulfide-isomerase A3 precursor	gi|45383890	56.18/5.76	2	281	↓	/	↓
19	SUCLG2	succinyl-CoA ligase [GDP-forming] subunit beta, mitochondrial	gi|57524960	46.76/6.49	4	282	↑	↑	/
21	DLD	dihydrolipoyl dehydrogenase, mitochondrial	gi|71897021	53.98/8.19	2	188	↑	/	/
22	DLST	dihydrolipoyllysine-residue succinyltransferase component of 2-oxoglutarate dehydrogenase complex, mitochondrial	gi|61098338	49.28/9.25	1	92	/	↑	/
23	ECH1	delta(3, 5)-Delta(2, 4)-dienoyl-CoA isomerase, mitochondrial-like, partial	gi|326936041	16.62/6.90	1	89	/	↓	/
24	ALDH6A1	methylmalonate-semialdehyde dehydrogenase [acylating], mitochondrial	gi|363734519	61.64/6.33	3	238	/	↓	↓
26	NDUFS8	NADH dehydrogenase [ubiquinone] iron-sulfur protein 8, mitochondrial	gi|118090950	23.82/5.84	3	247	/	↓	/
27	N/A	trypsinogen precursor	gi|242253868	25.88/6.85	1	127	/	↑	↑
28	PRDX3	thioredoxin-dependent peroxide reductase, mitochondrial	gi|363735594	25.76/8.58	2	190	/	↓	/
30	PCK2	phosphoenolpyruvate carboxykinase [GTP], mitochondrial precursor PEPCK	gi|45382653	70.97/8.16	5	240	/	↓	/
31	KRT1	unnamed protein product	gi|189054178	66.02/7.62	3	93	/	↑	/
32	GLUL	glutamine synthetase	gi|45382781	42.14/6.38	1	60	/	↑	/
33	LOC100655872	delta(3, 5)-Delta(2, 4)-dienoyl-CoA isomerase, mitochondrial-like	gi|344298379	35.70/8.09	1	107	/	↑	/
34	SDHA	succinate dehydrogenase [ubiquinone] flavoprotein subunit, mitochondrial-like	gi|326917263	72.54/6.85	6	302	/	↓	/
36	P4HB (PDI)	prolyl-4-hydroxylase (AA 5 - 494)	gi|63739	54.87/4.66	5	535	/	↑	/
37	SLC25A13	calcium-binding mitochondrial carrier protein Aralar2	gi|61098440	74.10/8.93	2	246	/	↑	↑
38	RGN	regucalcin	gi|45382019	33.23/5.77	1	160	/	/	↑
39	SUOX	Chain A, Sulfite Oxidase From Chicken Liver	gi|3212610	50.94/5.53	4	329	/	/	↑
40	TST	thiosulfate sulfurtransferase	gi|268370289	32.89/6.08	2	99	/	/	↑
41	CAT	hypothetical protein RCJMB04_1j22	gi|53127216	60.03/8.09	5	240	/	/	↑
42	PDHB	pyruvate dehydrogenase E1 component subunit beta, mitochondrial	gi|310750374	38.88/5.95	3	164	/	/	↑
43	CPOX	coproporphyrinogen-III oxidase, mitochondrial	gi|50729640	45.43/8.56	3	79	/	/	↑
44	LOC395933	sulfotransferase	gi|45384226	36.24/5.89	1	60	/	/	↓

^$^PMF: Peptide mass fingerprinting; pI: isoelectric point; MW: molecular weight.

^#^Compared with control group, ↑indicated up-regulated; ↓indicated down-regulated; /indicated no change.

**Table 2:** Differentially-expressed proteins in cytoplasm of liver in broiler chickens with (-)-HCA treatment identified by PMF query$.

Spot No.	Gene Ontology	Protein name	Accession No.	Exp. MW(KDa)/pI	Matched peptides	Score	Expression change treated with (-)-HCA#		
							1000 mg/Kg	2000 mg/Kg	3000 mg/Kg
1	PDXK	pyridoxal kinase	gi|363728772	35.01/5.93	3	347	↑	↑	↑
2	PGK	phosphoglycerate kinase	gi|45384486	44.72/8.31	3	275	↑	↑	↑
3	ARHGDIA	rho GDP-dissociation inhibitor 1	gi|124249432	23.27/5.22	2	159	↑	/	/
4	N/A	Chain A, Complex Of The Second Kunitz Domain Of Tissue Factor Pathway Inhibitor With Porcine Trypsin	gi|2914482	23.48/8.26	1	94	↑	↑	/
5	ALB	ALB protein	gi|74267962	69.24/5.88	2	153	↑	/	/
6	GART	trifunctional purine biosynthetic protein adenosine-3	gi|47825387	106.54/7.51	2	111	↑	↑	/
7	ADSL	adenylosuccinate lyase	gi|51874220	54.50/6.52	2	155	↑	/	/
8	ANXA5	Chain A, Crystal Structures Of Chicken Annexin V In Complex With Ca2+	gi|62738641	36.07/5.61	5	469	↑	/	↑
9	ALB	serum albumin precursor	gi|30794280	69.32/5.82	3	318	↑	↑	↑
10	MAT1A	S-adenosylmethionine synthase isoform type-1	gi|313760551	43.75/6.28	4	261	↑	/	/
11	HBG2	ORF	gi|212491	16.45/8.85	2	134	↓	↓	/
12	ME1	NADP-dependent malic enzyme	gi|45383538	62.00/6.45	1	66	↓	/	↓
13	TPI1	Chain A, Structure Of Triose Phosphate Isomerase From Chicken Muscle	gi|230359	26.54/7.26	2	130	↓	/	/
14	PFAS	phosphoribosylformylglycinamidine synthase	gi|334323278	144.35/5.36	1	60	↑	/	/
16	BHMT	betaine--homocysteine S-methyltransferase 1	gi|50755288	45.01/7.56	4	523	/	↑	/
17	PAH	phenylalanine-4-hydroxylase	gi|47604920	51.09/6.49	4	300	/	↑	/
18	PCBD1	pterin-4-alpha-carbinolamine dehydratase	gi|45382483	12.00/6.04	2	125	/	↑	/
19	ALB	serum albumin precursor	gi|30794280	69.32/5.82	2	142	↑	↑	↑
20	HMGCS2	hydroxymethylglutaryl-CoA synthase	gi|363736325	55.17/8.51	3	192	/	↑	/
22	CMBL	carboxymethylenebutenolidase homolog	gi|326917168	28.16/6.45	2	106	/	↓	/
23	BPNT1	3′(2′), 5′-bisphosphate nucleotidase 1	gi|308818127	32.91/5.60	1	143	/	↑	/
25	GNMT	glycine N-methyltransferase-like	gi|327265472	33.29/6.10	2	152	/	/	↓
27	CALR	calreticulin	gi|18389889	46.29/4.39	1	79	/	/	↑
28	TST	thiosulfate sulfurtransferase	gi|268370289	32.89/6.80	2	192	↑	↑	/
29	ECHS1	enoyl-CoA hydratase precursor	gi|15982640	28.25/8.72	1	67	↑	↑	↑
30	GAMT	guanidinoacetate N-methyltransferase	gi|363743812	23.74/5.56	3	200	/	/	↑

^$^PMF: Peptide mass fingerprinting; pI: isoelectric point; MW: molecular weight.

^#^Compared with control group, ↑ indicated up-regulated; ↓ indicated down-regulated; /indicated no change.
